# A Comparative Evaluation of Anesthetic Effectiveness of 4% Articaine vs 0.5% Bupivacaine for Lower Molar Tooth Extraction

**DOI:** 10.7759/cureus.32611

**Published:** 2022-12-16

**Authors:** Pavan Tenglikar, Abhigyan Manas, Amiya Ranjan Sahoo, Shreedevi Bhoi, Arundhati Singh, Prajakta B Patil, Anuradha B

**Affiliations:** 1 Department of Dentistry, College of Medicine, Komar University of Science and Technology, Sulaymaniyah, IRQ; 2 Department of Dentistry and Oral Surgery, Uttar Pradesh University of Medical Sciences, Saifai, IND; 3 Department of Oral and Maxillofacial Surgery, Awadh Dental College and Hospital, Jamshedpur, IND; 4 Department of Oral and Maxillofacial Surgery, Hazaribag College of Dental Sciences and Hospital, Hazaribagh, IND; 5 Department of Conservative Dentistry and Endodontic, Bharati Vidyapeeth Dental College and Hospital, Sangli, IND; 6 Department of Periodontics, Saveetha Dental College and Hospitals, Chennai, IND

**Keywords:** onset, bupivacaine, articaine, anesthesia, action

## Abstract

Background:Safe and efficient pain control is essential for today's dental practice. This randomized controlled study was conducted to evaluate the effectiveness of 0.5% bupivacaine with 4% articaine in lower molar tooth extraction.

Methods:One hundred subjects were classified into two groups, with 50 samples for each. Group A participants were managed with 0.5% bupivacaine with 1:200,000 epinephrine and group B participants with 4% articaine with 1:100,000 epinephrine for mandibular first and second molar extraction. Criteria such as onset and duration of anesthesia, pain throughout the procedure, pain during injection, and pain after the procedure were evaluated. Systolic and diastolic blood pressure (mmHg) and heart rate (per minute)were evaluated for all participants.

Results: There was a faster onset (53.2 vs 83.1 s) and lesser duration of action (216.6 vs 298.4 min) with articaine (group B) compared to bupivacaine (group A). Thirty-eight (76.0%) participants in group A and 44 (88.0%) participants in group B did not require re-anesthesia, whereas 12 (24%) participants in group A and six (12%) participants in group B required one-time re-anesthesia and it was insignificant.

Conclusion:Articaine has a faster onset but a relatively lower duration of action and requires statistically insignificant but lower re-anesthesia. As a result, articaine anesthesia can be efficiently recommended in oral surgical techniques.

## Introduction

Safe and efficient pain control is essential for today's dental practice. Pain is a subjective symptom that varies from person to person. Effective surgical procedures necessitate painless treatment, and local anesthesia (LA) is extensively used in dentistry to control pain. With LA administration, patient awareness remains unchanged, whereas nerve transmission is barred provisionally, definitively, and reversibly [1]. Lidocaine (lignocaine) is extensively used and considered the gold standard. It has a shorter duration of action and is safer in relation to other anesthetics. Articaine and bupivacaine are efficient and equivalent to lidocaine [2].

An idyllic LA delivers full sensory obstruction and should provide a satisfactory period of action. Collins et al. observed that bupivacaine is superior to lidocaine for quicker onset of action and an extended period of action. Lidocaine has a shorter duration of action and a slower onset of action, which makes search for alternative local anesthetic agents [3]. Several researchers have worked to check for an efficient local anesthetic agent with a quicker onset, lower complications, and reduced pain by altering the chemical and physical properties of LA [4]. To overcome the disadvantages of lidocaine, alternative local anesthetic agents, such as articaine and bupivacaine were tried [1-5]. Zhang et al. observed faster action with articaine compared to lidocaine during the third molar extraction procedure [5].

Articaine is an amide-type local anesthetic, and it has a benzene ring. It is an effective local anesthetic agent and it is presented as a 4% solution with a 1:100,000 epinephrine concentration. The existence of a thiophene ring in its structure represents it as a powerful agent amongst other local anesthetics. It is more soluble in lipids and is tolerated well by tissues. It can be used for peripheral nerve blocks or local infiltration [6]. It has been perceived from previous research that amongst numerous local anesthetic agents, articaine is observed to be relatively rapid-acting, harmless, and appropriate for oral surgical practice [1,4,6-8].

The preference for local anesthetic solutions in tooth extraction depends on the following three important scientific concerns: latency, anesthetic effectiveness, and the duration of the anesthetic effect and also on preexisting pain/local inflammation/hot tooth, etc. [9]. The present comparative research was done to assess the effectiveness of 0.5% bupivacaine with 4% articaine in oral lower molar extraction based on objectives, such as duration of anesthesia, onset, and pain perception during the oral surgical procedure and also to check changes in blood pressure and heart rate.

## Materials and methods

Study design

This randomized controlled research was performed in the oral surgery department from June 2017 to October 2019. The study comprises 100 patients of both sexes who visited the oral surgery department for lower molar tooth extraction in the age range of 20-50 years. Group A had 30 males and 20 females, while group B had 27 males and 23 female participants. Before start of the procedure, a null hypothesis was planned that there was no difference between the articaine and bupivacaine groups in their mechanism of action.

Inclusion criteria and exclusion criteria

Conditions for inclusion were as follows: systemically healthy participants whose ages ranged from 20 to 50 years and lower molar teeth indicated for extraction. An exclusion condition includes patients aged below 20 years, pregnant or lactating women, people with an allergic history to local anesthetic solutions, disobliging patients and preexisting conditions, painful teeth, etc.

Ethical approval for the study

Ethical consent for the research was attained from the Institutional Review Board (IRB) of Awadh Dental College and Hospital, Jharkhand, India with ref no. ADCH/2017-0042. Informed agreement was attained from all the participating subjects. The study procedures adhered to the Declaration of Helsinki's ethical guidelines.

Sample size estimation

Patient’s demographic outline was recorded. The sample size was determined based on the fact that the success rate of local anesthetic solutions ranges between 90% and 95%. Therefore, supposing p=90 as the frequency of achievement rate with a 9% margin of error, the formula used was n=Z^2^_α__/2_ pq/d^2^, where p is success rate, q=1-p, d is the margin of error, and Z_α/2_ is the ordinate of standard normal distribution at α% level of significance. Hence total sample size of 100 was selected.

Study procedure

Samples were distributed randomly based on the lottery method and who met the inclusion criteria with double-blinding procedure as group A with bupivacaine group and group B with articaine group. The study was done by a single trained investigator. Patients were categorized into groups A and B, with 50 samples in each group. Group A was given a 1:200,000 epinephrine with 0.5% bupivacaine injection (Livealth BioPharma Pvt. Ltd., India), while group B was given a 1: 100,000 epinephrine with 4% articaine HCl injection (Septocaine®, Septodont Inc., Canada).

Prior to the study, the investigator assigned the arrangement of subjects’ identification numbers to group A as bupivacaine group and group B as articaine group with 50 samples in each group. Patients necessitating surgical extraction of mandibular first or second molar teeth were delivered with 1.5 mL of anesthetic solution in both groups to anesthetize the inferior alveolar nerve, buccal, and lingual nerve. A single trained investigator performed all extractions and anesthesia using aseptic standard surgical procedures. Evaluation for anesthetic effects pertaining to pain during injection, onset and duration of anesthesia, pain throughout the technique, and after the technique was performed and recorded by the same trained investigator [8,9]. Systolic and diastolic blood pressure (mmHg), and heart rate (per minute) were evaluated for all participants during and postoperative period. Subsequent to extraction, patients were put on analgesic and antibiotic coverage for five days.

Evaluation

The length of the surgical method and the period of postoperative anesthesia and pain were measured as mentioned - the injection time to the patient's first indication of numbness was used to determine the start of anesthesia [8,9]. Both subjective (absence of sensitivity to the lower lip, half of the tongue, and the buccal mucosa) and objective symptoms were tested at the start of the anesthetic agent (by probing or pressure for onset of anesthesia around the gingival tissues). Pain evaluations during injection and the effectiveness of anesthesia can be predicted once the extraction is done by means of the visual analog scale (VAS), where 0 indicates absence of pain and 10 indicates severe pain, and the period of surgery after anesthetic injection was measured by noting the onset timing of anesthesia and the absence of numbness on the soft tissues (mucosa, tongue, and lower lip) afterward for duration of soft tissue anesthesia. The time between the conclusion of the procedure and the first ibuprofen (Brufen-400, Abbott, India) tablet taken for pain relief was used to calculate the duration of postoperative analgesia (in minutes) as mentioned by a patient.

Statistical analysis

The obtained data were tabulated and statistically evaluated with SPSS version 20.0 (SPSS Inc., Chicago, IL) using the chi-square test and t‑test with p<0.05.

## Results

Table 1 indicates the assessment of various parameters in both groups. The mean onset of local anesthetic action in group A was 83.1±13.3 s and in group B it was 53.2±5.8 s. The length of soft tissue anesthesia in group A was 298.4±25.7 min and in group B it was 216.6±27.6 min. The duration of the postoperative analgesic effect was 210.7±3.67 min in group A and 196.1±10.4 min in group B. Systolic blood pressure, diastolic blood pressure, and heart rate (per min) was non-significant among the groups, during and postoperative period. Pain score assessment using VAS was found to be insignificant in both groups (Table 2).

**Table 1:** Assessment of various parameters for bupivacaine and articaine groups. The test used was t-test. *P-value <0.05 was considered significant.

Parameter		Group A bupivacaine		Group B articaine		p-Value*
		Mean	SD	Mean	SD	
Beginning of action (seconds)		83.1	13.3	53.2	5.8	0.02
Duration of soft tissue anesthesia (minutes)		298.4	25.7	216.6	27.6	0.01
Duration of postoperative analgesic effect (minutes)		210.7	3.67	196.1	10.4	0.71
Systolic blood pressure (mmHg)	During procedure	123.3	-	124.5	-	0.43
	Postoperatively	122.2	-	123.3	-	0.49
Diastolic blood pressure (mmHg)	During procedure	73.3	-	72.6	-	0.65
	Postoperatively	74.2	-	73.5	-	0.62
Heart rate (per minute)	During procedure	81.3	-	80.5	-	0.62
	Postoperatively	81.5	-	80.7	-	0.58

**Table 2:** Assessment of pain score with VAS. VAS: visual analog scale *P-value <0.05 was considered significant.

Groups	Group A bupivacaine		Group B articaine		t-test	p-Value*
	Mean	SD	Mean	SD		
Pain while delivery of anesthesia (mm)	3.46	2.11	5.03	3.51	0.738	0.35
Pain score postoperatively (mm)	10.56	4.12	12.48	5.65	-	0.46

Table 3 indicates that 38 (76.0%) participants in group A and 44 (88.0%) participants in group B did not need re-anesthesia, whereas 12 (24%) participants in group A and six (12%) participants in group B needed one-time re-anesthesia. The chi-square test showed non-significant alteration among both groups (p>0.05). In our study, it was observed that the necessity of re-anesthesia was less with articaine compared to bupivacaine.

**Table 3:** Assessment of requirement for re-anesthesia. The test used was chi-square test. *P-value <0.05 was considered significant.

Need for re-anesthesia	Group A bupivacaine	Group B articaine	p-Value*
	Frequency	Frequency	
No need for re-anesthesia	38 (76%)	44 (88%)	0.21
Requiring re-anesthesia	12 (24%)	6 (12%)	0.32

## Discussion

Bupivacaine is frequently selected due to its longer duration of analgesia and postoperative pain control. Thakare et al. observed that the bupivacaine group demonstrated constant pressure sensation and uneasiness compared to the articaine group [2].

The effectiveness of an anesthetic agent can be judged by its capacity to reduce pain, the time taken for the onset of action, and the extended period of anesthetic effect. Lignocaine is usually recognized as “lidocaine,” which is an amide type of local anesthetic agent with a shorter duration of action [7]. The World Health Organization (WHO) has included lignocaine in its necessary drug list. It displays its properties by blocking nerve fiber impulses [10]. Articaine also conveys its action comparable to lidocaine by binding to voltage-gated sodium channels and inhibiting the influx of sodium ions [11].

In a systematic review, Bhattarai et al. stated that bupivacaine demonstrated better anesthetic and analgesic efficacy but poor onset of action in contrast to other local anesthetic medicaments evaluated for oral surgical procedures, similar to our findings [12]. Badr and Aps concluded from their review that not a single dental local anesthetic agent (lidocaine, 0.5% bupivacaine, 3% mepivacaine, 4% articaine, and 0.75% levobupivacaine) provided 100% anesthesia and efficient technique required during tooth extraction [13]. Brajkovic et al. assessed the efficiency of levobupivacaine over bupivacaine for third molar extraction and found that 0.5% levobupivacaine was superior to 0.5% bupivacaine in terms of intensity and longevity of postoperative analgesia and intraoperative anesthesia [14]. Sancho-Puchades et al. evaluated the effectiveness of articaine over bupivacaine for third molar removal and stated that bupivacaine showed considerably longer-lasting soft tissue anesthesia, and it is effective over articaine because of its quick postoperative pain prevention capacity [15]. The report is in contrast to our results. We found a longer duration of anesthetic action with bupivacaine, but the onset of action was faster with articaine. Adelusi et al. evaluated the effectiveness of lidocaine over bupivacaine and found higher patient satisfaction for bupivacaine [16]. Pellicer-Chover et al. evaluated the effectiveness of bupivacaine over articaine for extraction of third molars. They concluded that articaine had higher clinical effectiveness compared to bupivacaine with lesser bleeding, latency time, and duration of soft tissue anesthesia [17]. Tokuç and Coşkunses also found greater efficacy with articaine compared to bupivacaine [18]. Similar results are found in the present study.

Present research assessed the effectiveness of 0.5% bupivacaine with 4% articaine in the extraction of molar teeth with the following objective criteria: pain during injection, duration and beginning of action of anesthesia, pain throughout the procedure, and pain following the procedure. It was observed in our study that there was a faster onset and moderate length of action with articaine (group B) compared to bupivacaine (group A). The duration of anesthesia was better with bupivacaine compared to articaine. Systolic blood pressure, diastolic blood pressure, and heart rate (per min) findings were statistically non-significant among the groups during and postoperative period.

When the pKa of local anesthetic agents is close to the pH of the tissue, where they are injected, anesthesia takes effect more quickly. The tissue, bupivacaine, and articaine had pKa values of 7.4, 8.1, and 7.8, respectively. As a result, articaine produces more free molecules in the tissue and diffuses into the nerve membrane more effectively [18]. It's possible that the rapid metabolism of articaine, which results from its hydrolysis-induced degradation, increased the gap between the postoperative anesthesia durations of bupivacaine and articaine in the current study. Because it is metabolized in the liver and plasma, articaine is eliminated more quickly than bupivacaine. As a result, articaine's postoperative analgesia lasts for a shorter duration than bupivacaine [18]. One of the reasons for longer duration of anesthetic effect of bupivacaine is its 1:200,000 epinephrine content compared to 4% articaine with 1:100,000 epinephrine.

Our study compared the postoperative efficacy of two local anesthetic agents using the VAS. The present study found that pain while the delivery of anesthesia and postoperative pain score (VAS) were comparable between articaine and bupivacaine (Table 2). Bupivacaine had a longer duration of action. The necessity of re-anesthesia was comparatively less with articaine than bupivacaine, but it was statistically insignificant. Our results and previous studies indicate that articaine is more effective than 0.5% bupivacaine in recovery from anesthesia. Henceforth, it can be suggested for tooth removal and other oral surgical techniques.

The limitations of the present study are smaller sample size and a single-center analysis. Anesthetic efficacy was based on the rescue analgesia taken by the patient at his or her discretion and lacks any objectivity. Further, randomization was lottery-based, and blinding was not very proper.

Future research should be directed to evaluate the efficacy of articaine with other anesthetic agents for other oral surgical procedures and pulp therapy on larger sample sizes, objective parameter-based rescue analgesia, and timing assessment.

## Conclusions

Both bupivacaine and articaine are effective anesthetic agents. However, articaine has a faster onset but a relatively lower duration of action and requires statistically insignificant but lower re-anesthesia. The systolic and diastolic blood pressure and heart rate were non-significant among the groups, during and after the procedure. The necessity of re-anesthesia was comparatively less with articaine compared to bupivacaine. Articaine is effective in anesthetizing molars during extraction.
